# Artificial Intelligence–Enabled Orthodontic Care for Remote and Underserved Populations: A Scoping Review of Access, Technology, and Public Health Integration

**DOI:** 10.1155/ijod/3557207

**Published:** 2026-09-12

**Authors:** Pruthvi Shetty, Shravan Shetty, Christal Varghese

**Affiliations:** ^1^ Department of Public Health Dentistry, AJ Institute of Dental Sciences and Hospital, NH-66 Kuntikana, Mangalore 575004, Karnataka, India; ^2^ Department of Orthodontics and Dentofacial Orthopaedics, Manipal College of Dental Sciences Mangalore, Manipal Academy of Higher Education, Manipal 576104, Karnataka, India, manipal.edu

**Keywords:** artificial intelligence, deep learning, machine learning, orthodontics, teledentistry

## Abstract

**Background:**

Unequal access to orthodontic care remains a major public health challenge, particularly in rural and underserved regions, recent advancements in artificial intelligence (AI) and digital orthodontics offer potential to bridge these gaps through remote diagnosis and monitoring.

**Aim:**

This scoping review aims to systematically map and categorize how AI applications in orthodontic diagnosis, treatment planning, and remote monitoring enhance access to care for underserved and remote populations while identifying specific access‐related outcomes, research gaps, and policy implications for public health integration.

**Methods:**

This scoping review followed the Arksey and O’Malley framework and was reported in accordance with the preferred reporting items for systematic reviews and meta‐analyses extension for scoping reviews (PRISMA‐ScR) guidelines. A comprehensive search was conducted across PubMed, Scopus, Embase, and Web of Science, supplemented with relevant gray literature. Studies published between January 2000 and September 2025 that discussed the use of AI in orthodontic diagnosis, treatment planning, appliance design, or teledentistry‐based service delivery were included. Two independent reviewers performed screening and data extraction using Rayyan, and results were synthesized descriptively.

**Results:**

A total of 23 studies met the inclusion criteria. The evidence indicated that AI‐assisted orthodontic systems can enhance diagnostic precision and reduce clinical workload. Remote monitoring platforms were shown to reduce in‐person appointments while maintaining clinical standards and improving patient compliance. However, majority of these studies were conducted in urban or institutional environments, highlighting a significant gap in real‐world longitudinal data for rural or low‐resource settings.

**Conclusion:**

While AI holds transformative potential to decentralize orthodontic care, current research remains largely limited to urban settings, necessitating a shift towards real‐world validation and ethical policy effort to ensure equitable delivery in underserved regions.

## 1. Introduction

Access to orthodontic care remains deeply unequal across the globe, particularly in rural and underserved regions where socioeconomic, geographic, and infrastructural barriers restrict access to specialized oral healthcare [1–3]. While dental caries and periodontal disease are more commonly discussed in the context of public health, malocclusion and orthodontic needs are often overlooked despite their significant psychosocial and functional impacts [4]. In many low‐resource or geographically isolated communities, orthodontic services are concentrated mainly in urban centers, creating disparities in early diagnosis, interceptive treatment, and continuity of care [5, 6].

Recent advancements in artificial intelligence (AI), digital orthodontics, and teledentistry have introduced transformative potential to mitigate these access barriers [7–9]. AI has shown promise in automating diagnostic procedures, improving treatment planning accuracy, and facilitating real‐time monitoring of patients through digital interfaces [10, 11]. By integrating AI with cloud‐based telecommunication and remote monitoring platforms, it may be possible to deliver orthodontic care beyond traditional clinic boundaries, allowing virtual supervision of treatment progress even in remote or resource‐constrained settings [12, 13].

The public health relevance of AI in orthodontics lies in its capacity to decentralize specialized care, reduce dependence on in‐person consultations, and empower community‐based health models where initial screening or data collection can occur remotely. AI‐driven mobile applications and automated cephalometric analyses can potentially reduce diagnostic delays and direct timely referrals, while AI‐enabled remote monitoring systems such as *Dental Monitoring* may extend orthodontic oversight without frequent clinical visits [14–17].

Despite this promise, the integration of AI into orthodontic public health delivery systems remains fragmented. Variability exists in algorithmic validation, interoperability of software platforms, and cost‐effectiveness across different populations [18, 19]. Furthermore, ethical and regulatory concerns, including data privacy, informed consent, and bias in AI models, continue to challenge its large‐scale implementation in clinical and community settings [20, 21].

To date, no comprehensive synthesis has mapped how AI technologies have been utilized to extend orthodontic care to underserved or remote populations. Existing reviews have focused primarily on clinical accuracy, diagnostic performance, or patient outcomes in urban and controlled environments, overlooking equity and access dimensions [22, 23]. This scoping review aims to map and categorize how AI application in orthodontic diagnosis, treatment planning, and remote monitoring improve access to care for underserved or remote population while identifying research gaps and policy implications for public health and teledentistry integration.

## 2. Methods

This scoping review was conducted according to the Arksey and O’Malley [24] framework and was reported following the preferred reporting items for systematic reviews and meta‐analyses extension for scoping reviews (PRISMA‐ScR) guidelines [25]. The review protocol was prospectively registered on the Open Science Framework under the registration number: https://osf.io/4d5f8. This scoping review was conducted to answer the research question: “How has artificial intelligence been applied in orthodontic diagnosis, treatment planning, and remote patient monitoring to improve access to orthodontic care among underserved or remote populations?”

### 2.1. Information Sources

Comprehensive search strategies were formulated using medical subject headings (MeSH) and free text encompassing AI, machine learning, and teledentistry with orthodontics. The systematic search spanned PubMed, Scopus, Embase, and Web of Science supplemented by a gray literature review via Google Scholar to ensure maximum coverage. Two independent reviewers conducted the search covering literature published between January 2000 and September 2025. Detailed search protocols, including specific Boolean operators and keywords utilized for each database, are provided in Table 1.

**Table 1:** Search strategies used across PubMed, Scopus, Embase, and Web of Science.

PubMed	(“Artificial Intelligence”[Mesh] OR “Machine Learning”[Mesh] OR “Deep Learning”[Mesh] OR “artificial intelligence”[tiab] OR “machine learning”[tiab] OR “deep learning”[tiab] OR “neural network ^∗^“[tiab] OR “AI”[tiab]) AND (“Orthodontics”[Mesh] OR orthodontic ^∗^[tiab] OR malocclusion ^∗^[tiab] OR “aligner therapy”[tiab] OR “tooth movement”[tiab] OR “treatment planning”[tiab]) AND (“Teledentistry”[Mesh] OR teledentistry[tiab] OR “tele‐orthodontic ^∗^”[tiab] OR “remote consultation”[tiab] OR “remote monitoring”[tiab] OR “rural health”[Mesh] OR “underserved”[tiab] OR “remote area ^∗^”[tiab] OR “low‐resource setting ^∗^”[tiab] OR “access to care”[tiab] OR “public health”[tiab]) AND (“2000/01/01”[Date ‐ Publication]: “2025/09/30”[Date ‐ Publication]) AND (english[lang])
Embase	(’artificial intelligence’:ti,ab OR ‘machine learning’:ti,ab OR ‘deep learning’:ti,ab OR ‘neural network ^∗^’:ti,ab OR ‘convolutional network ^∗^’:ti,ab OR AI:ti,ab) AND (Orthodontics/exp OR orthodontic ^∗^:ti,ab OR malocclusion ^∗^:ti,ab OR ‘aligner therapy’:ti,ab OR ‘tooth movement’:ti,ab OR ‘treatment planning’:ti,ab) AND (Teledentistry/exp OR teledentistry:ti,ab OR tele‐orthodontic ^∗^:ti,ab OR ‘remote consultation’:ti,ab OR ‘remote monitoring’:ti,ab OR ‘rural health’:ti,ab OR underserved:ti,ab OR ‘remote area ^∗^’:ti,ab OR ‘low‐resource setting ^∗^’:ti,ab OR ‘access to care’:ti,ab OR ‘public health’:ti,ab)
Scopus	(TITLE‐ABS(“artificial intelligence”) OR TITLE‐ABS(“machine learning”) OR TITLE‐ABS(“deep learning”) OR TITLE‐ABS(“neural network ^∗^”) OR TITLE‐ABS(“convolutional network ^∗^”) OR TITLE‐ABS(AI)) AND (INDEXTERMS(Orthodontics) OR TITLE‐ABS(orthodontic ^∗^) OR TITLE‐ABS(malocclusion ^∗^) OR TITLE‐ABS(“aligner therapy”) OR TITLE‐ABS(“tooth movement”) OR TITLE‐ABS(“treatment planning”)) AND (INDEXTERMS(Teledentistry) OR TITLE‐ABS(teledentistry) OR TITLE‐ABS(tele‐orthodontic ^∗^) OR TITLE‐ABS(“remote consultation”) OR TITLE‐ABS(“remote monitoring”) OR TITLE‐ABS(“rural health”) OR TITLE‐ABS(underserved) OR TITLE‐ABS(“remote area ^∗^”) OR TITLE‐ABS(“low‐resource setting ^∗^”) OR TITLE‐ABS(“access to care”) OR TITLE‐ABS(“public health”))
Web of Science	(“artificial intelligence” OR “machine learning” OR “deep learning" OR “neural network ^∗^” OR “convolutional network ^∗^” OR AI) AND (Orthodontics OR orthodontic ^∗^ OR malocclusion ^∗^ OR “aligner therapy” OR “tooth movement” OR “treatment planning”) AND (Teledentistry OR teledentistry OR tele‐orthodontic ^∗^ OR “remote consultation” OR “remote monitoring” OR “rural health” OR underserved OR “remote area ^∗^“ OR “low‐resource setting ^∗^” OR “access to care” OR “public health”)

### 2.2. Eligibility Criteria

The review followed the population–concept–context (PCC) framework as recommended by the Joanna Briggs Institute [26]. The population included patients receiving orthodontic care in remote, underserved, or low‐resource settings, as well as dental professionals and AI developers. The concept focused on AI applications (machine learning, deep learning, and neural networks) in diagnosis, treatment planning, and remote monitoring systems. The contest encompassed studies emphasizing teledentistry and public health integration. We included peer‐reviewed original research, clinical trials, and narrative reviews that provided technical or public‐health synthesis of AI in orthodontics, published between January 2000 and September 2025. Exclusion criteria were applied to nonorthodontic AI applications, editorials, and opinion pieces.

### 2.3. Study Selection

**Figure 1:**
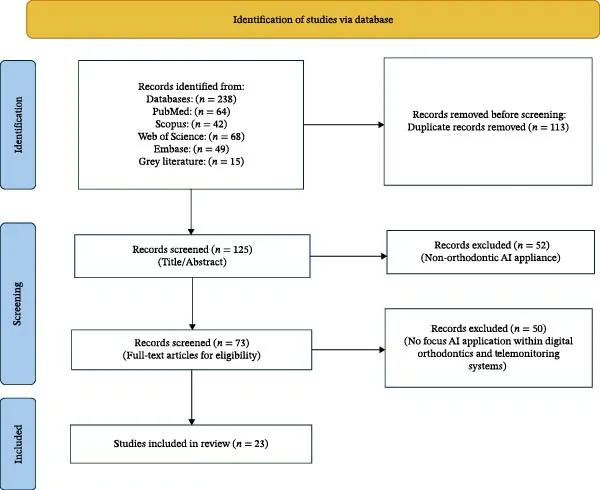
PRISMA chart.

The search results were managed using Mendeley software version 2.77.0 Mendeley Ltd. The screening process was conducted in two distinct phases using Rayyan software. The first two reviewers (Pruthvi Shetty and Shravan Shetty) independently screened the titles and abstracts against the inclusion criteria. Subsequently, the same reviewers subsequently performed a full‐text assessment to determine the final eligibility. Any disagreements or discrepancies throughout the selection process were resolved through discussion with the third author (Christal Varghese) until a consensus was reached. The final selection consisted of 23 studies, as illustrated in the PRISMA flow diagram (Figure 1).

### 2.4. Data Extraction

A standardized data charting form was developed to systematically organize and capture key variables from the included studies. The form was jointly designed and iteratively updated by two reviewers (Pruthvi Shetty and Shravan Shetty), with the third author (Christal Varghese) resolving any discrepancies. The final data set was verified by all three authors to ensure accuracy and achieve mutual consensus.

Synthesis of results—the findings were synthesized descriptively to account for the heterogeneity in study methodologies. Thematic analysis was performed involving multiple readings of the selected articles by two researchers (Pruthvi Shetty and Shravan Shetty), later refined through discussion with the third researcher (Christal Varghese). The analysis prioritized technological utility and accessibility impact within three primary domains: AI in initial diagnosis and screening, AI‐driven remote patient monitoring, and integration into public health frameworks. The Crowe Critical Appraisal Tool (CCAT) was employed to evaluate the quality of the original research studies included in the review. Each article was assessed across eight categories, with scores ranging from 0 to 5 per category for a maximum total score of 40. On the bases of these scores, studies were classified as high quality (>35), medium quality (25–34), or low quality (<25) (Table 2).

**Table 2:** Quality appraisal of included studies (CCAT).

Author and year of publication	Preliminaries	Introduction	Design	Sampling	Data collection	Ethical	Results	Discussion	Total	Quality of study
Snider et al., 2023 [5]	4	5	5	4	4	4	4	4	34	Medium
Homsi et al., 2023 [6]	4	5	5	4	5	5	5	3	36	High
Surovková et al., 2023 [7]	4	5	3	3	4	4	4	2	29	Medium
Albertina et al., 2023 [16]	4	4	3	3	3	2	3	3	25	Medium
Snider et al., 2024 [4]	4	5	5	3	4	5	5	3	34	Medium
Kavousinejad et al., 2024 [21]	3	3	2	3	3	2	2	2	20	Low
Kılıç et al., 2024 [10]	5	4	4	4	4	5	4	4	34	Medium
Sharma et al., 2025 [1]	4	4	3	3	4	5	3	4	30	Medium
Elnagar et al., 2025 [3]	4	5	5	4	5	5	5	1	34	Medium

## 3. Results

The systematic search across databases and gray literature initially identified 238 records. After removing duplicates and conducting a two‐stage screening process, 23 studies met the inclusion criteria for final synthesis. Notably, while the search strategy spanned from January 2000 to September 2025, all included articles are published between 2021 and 2025. This temporal concentration highlights recent and rapid surge of interest in AI application within digital orthodontics and telemonitoring systems. The data obtained were summarized under the following headings: bibliographic information (authors, journal), study design and methodology, type of AI technology and orthodontic application, population and setting characteristics, key findings related to diagnosis, treatment planning, remote monitoring, and public health or access‐related outcomes, which is provided as Table S1 (see Supporting Information).

### 3.1. Thematic Analysis

#### 3.1.1. AI in Initial Diagnosis and Screening for Underserved Populations

A recurring theme across several studies was the use of AI in automated diagnosis and early screening, addressing one of the most significant access barriers in orthodontic care. AI algorithms demonstrated competence in automated cephalometric landmark detection and skeletal pattern recognition, reducing operator dependency and improving diagnostic accuracy [10, 12, 14]. Machine learning–based mobile applications provide face‐centered screening tools that allow image‐based skeletal classification, even in the absence of radiographic equipment [10].

Such tools are particularly beneficial for underserved populations, where early interceptive orthodontic care is often delayed. Automated diagnostic support using mobile AI platforms has the potential to triage cases requiring specialist attention, optimizing specialist availability and reducing unnecessary travel [14, 23].

#### 3.1.2. Remote Patient Monitoring to Overcome Geographical Barriers

The second dominant theme centered on AI‐driven remote monitoring (AIRM), with *Dental Monitoring* emerging as the most extensively validated platform [1, 4–7, 9, 17]. AI was employed to analyze intraoral images or videos captured through mobile applications, enabling continuous assessment of tooth movement, appliance wear compliance, and oral hygiene maintenance [4–6].

Studies have demonstrated that remote monitoring significantly reduces in‐person appointment frequency, decreases treatment time, and maintains comparable clinical outcomes relative to conventional in‐office supervision [3, 5, 6]. For patients in remote or underserved areas, these systems enhance accessibility while preserving quality standards of care. Furthermore, AI algorithms were shown to detect oral hygiene lapses, such as plaque accumulation, and generate real‐time reminders, thereby improving patient compliance and outcomes [1, 4, 5].

#### 3.1.3. Public Health Context and Future Integration

Several studies discussed the potential role of AI‐enabled teledentistry within a broader public health framework. AI‐supported teleorthodontic systems were highlighted as having the potential to improve access to orthodontic care, particularly for rural, geographically remote, and mobility‐restricted populations. However, direct evidence from underserved or low‐resource settings remains limited. Several authors proposed integration into primary healthcare centers or mobile dental units as a potential strategy for improving orthodontic access. Nevertheless, important limitations remain. Few studies reported large‐scale validation or cost‐effectiveness analyses, and inadequate technological infrastructure continues to represent a barrier in resource‐limited settings [21, 23]. Ethical issues such as data privacy, algorithmic transparency, and limited diversity in AI training datasets were frequently identified as important challenges, as these factors may reduce the generalizability of AI systems when applied to underserved populations [19, 21].

## 4. Discussion

This scoping review, conducted according to the Arksey and O’Malley framework [24] and PRISMA‐ScR guidelines [25], mapped a rapidly evolving landscape of AI applications in orthodontics. By applying the CCAT [27], we identified that while technical efficacy of AI is high, the evidence remains concentrated in urban, high resource settings.

Diagnostic automation and screening represent the first pillar of improved access. Kavousinejad et al. [21] demonstrated how deep learning frameworks can automate the classification of diagnostic images with 99% accuracy, while Kılıç et al. [10] highlighted a mobile application capable of diagnosing skeletal malocclusion from profile photographs without radiographs. Such tools facilitate community level triage in underserved areas where specialist availability is limited. Narrative reviews by Moeini and Torabi [14], Gao et al. [19], and Surdu et al. [23] further emphasize that AI systems reduce interexaminer variability and accelerate workflows, which is essential for large‐scale public health screenings.

The second domain involves treatment planning and digital workflow optimization. Albertina et al. [16] reported that AI‐driven software significantly improves the efficiency of clear aligner therapy compared to conventional methods. Olawade et al. [2] and Van Liedekerke et al. [13] reported that AI predictive systems can reduce overall treatment time by several months, while Chu and Ooi [18] highlighted that such digital workflows enhance patient communication and predictability, despite challenges like high equipment cost and learning curves.

Patient AIRM is the most robustly evidence domain, yet it faces significant commercial concentration. Among the included studies, nearly all included Dental Monitoring platform. Sharma et al. [1] and Snider et al. [4, 5] stated that this technology effectively improves oral hygiene and reduces plaque indices through AI‐based active notification. Furthermore, Elnagar et al. [3] and Homsi et al. [6] confirm that AI‐driven tooth tracking is clinically accurate and usable for remote supervisions. Reviews by Almishkha et al. [11], Bijoy et al. [15], and Polizzi et al. [22] suggest that these platforms can reduce the number of in‐person visits, a crucial factor for rural population facing geographical barriers. Snider et al. [5] caution that AI still tends to underreport certain hygiene conditions, and Squires et al. [17] raised ethical concerns regarding data privacy and professional‐patient relationship.

Finally, the ethical and policy implications of AI integration are paramount. As Lognay and Noortgate [12], Van Liedekerke et al. [13], and Kaushik and Rapaka [20] observed, the potential for algorithm bias exists if training datasets are not representative of diverse population. To ensure that AI promotes oral health equity rather than widening the digital divide, future implementation must align with the WHO global oral health action plan 2023–2030 [28], focusing on sustainable public health models rather than solely commercial urban applications.

### 4.1. Limitations of the Evidence Base

First, the current evidence base is predominantly derived from urban and institutional settings, consisting largely of proof‐of‐concept studies with limited longitudinal evaluation of access‐related outcomes, such as reductions in travel burden, treatment costs, or service availability in rural and underserved communities. Second, quality appraisal was restricted to the nine original research studies because an appropriate validated appraisal tool was not applied to narrative reviews and other nonprimary evidence sources. Consequently, the findings derived from these studies should be interpreted with appropriate caution. Furthermore, the inclusion of one low‐quality primary study may have influenced the overall synthesis, although its findings were interpreted in the context of the broader evidence base. Third, the available evidence is heavily concentrated on a single commercial platform (Dental Monitoring), which may limit the generalizability of the findings to other AI‐enabled remote monitoring systems and the broader field of AI‐assisted orthodontics.

## 5. Conclusion

While AI has emerged as a transformative enabler with the potential for decentralized care, current research is largely limited to urban settings. To truly bridge the orthodontic access gap, future research must shift from preliminary validation to large‐scale implementation in underserved regions. Policy efforts should focus on overcoming infrastructural barriers and establishing ethical, bias‐resistant framework to ensure that AI‐driven orthodontics promotes global oral health equity rather than widening the digital divide.

**Nomenclature**
AI: Artificial intelligence
AIRM: AI‐driven remote monitoring
CCAT: Crowe Critical Appraisal Tool
MeSH: Medical subject headings
PCC: Population, concept, context
PRISMA‐ScR: Preferred reporting items for systematic reviews and meta‐analysis extension for scoping reviews.

## Author Contributions

**Pruthvi Shetty**: conceptualization, methodology, investigation, data curation, formal analysis, writing – original draft. **Shravan Shetty**: conceptualization, methodology, investigation, data curation, formal analysis, supervision, validation, writing – review and editing. **Christal Varghese:** methodology, investigation, data curation, formal analysis, writing – review and editing.

## Funding

This study did not receive any specific funding.

## Disclosure

All the authors have read and approved the final version of the manuscript. Shravan Shetty (corresponding author) had full access to all of the data in this study and takes complete responsibility for the integrity of the data and the accuracy of the data analysis.

## Ethics Statement

Institutional ethics approval was not needed for this study as it is the scoping review of previously published literature and did not involve direct contact with the human participants or animals or the use of primary raw patient data. The ethical reporting and oversight of included primary research studies were evaluated as a specific criterion during the quality appraisal process using the Crowe Critical Appraisal Tool (CCAT).

## Conflicts of Interest

The authors declare no conflicts of interest.

## Supporting Information

Additional supporting information can be found online in the Supporting Information section.

## Supporting information


**Supporting Information** Table S1: Data extraction and charting of included studies. This table provides comprehensive bibliographic details, study designs, specific AI technology applications, and detailed public health/access‐related outcomes for all included studies.

## Data Availability

The authors confirm that the data supporting the findings of this study are available within the article or its Supporting Information.

## References

[1] Sharma A. , Sharma T. , and Tomer G. , et al.Assessment of AI-Based Monitoring Method in Assessing Oral Hygiene During Orthodontic Treatment, Journal of Orthodontic Science. (2025) 14, 17. 40630780 10.4103/jos.jos_146_24 PMC12237006

[2] Olawade D. B. , Leena N. , and Egbon E. , et al.AI-Driven Advancements in Orthodontics for Precision and Patient Outcomes, Dentistry Journal. (2025) 13, no. 5, 10.3390/dj13050198, 198. 40422618 PMC12110745

[3] Elnagar M. H. , Nieberg-Baskin H. , and Becher M. K. , et al.Assessing an AI-Driven Remote Monitoring System for Reliable Clinical Photo Acquisition and Patient usability, Journal of the World Federation of Orthodontists. (2025) 14, no. 6, 344–354, 10.1016/j.ejwf.2025.08.002. 41006147

[4] Snider V. , Homsi K. , and Kusnoto B. , et al.Clinical Evaluation of Artificial Intelligence Driven Remote Monitoring Technology for Assessment of Patient Oral Hygiene During Orthodontic Treatment, American Journal of Orthodontics and Dentofacial Orthopedics. (2024) 165, no. 5, 586–592, 10.1016/j.ajodo.2023.12.008. 38363256

[5] Snider V. , Homsi K. , and Kusnoto B. , et al.Effectiveness of AI-Driven Remote Monitoring Technology in Improving Oral Hygiene During Orthodontic Treatment, Orthodontics & Craniofacial Research. (2023) 26, no. S1, 102–110, 10.1111/ocr.12666. 37113065

[6] Homsi K. , Snider V. , and Kusnoto B. , et al.In-Vivo Evaluation of Artificial Intelligence Driven Remote Monitoring Technology for Tracking Tooth Movement and Reconstruction of 3-Dimensional Digital Models During Orthodontic Treatment, American Journal of Orthodontics and Dentofacial Orthopedics. (2023) 164, no. 5, 690–699, 10.1016/j.ajodo.2023.04.019. 37341668

[7] Surovková J. , Haluzová S. , Strunga M. , Urban R. , Lifková M. , and Thurzo A. , The New Role of the Dental Assistant and Nurse in the Age of Advanced Artificial Intelligence in Telehealth Orthodontic Care With Dental Monitoring: Preliminary Report, Applied Sciences. (2023) 13, no. 8, 10.3390/app13085212, 5212.

[8] Park J. H. , Rogowski L. , Kim J. H. , Al Shami S. , and Howell S. E. I. , Teledentistry Platforms for Orthodontics, Journal of Clinical Pediatric Dentistry. (2021) 45, no. 1, 48–53, 10.17796/1053-4625-45.1.9. 33690830

[9] Park J. H. , Kim J. H. , Rogowski L. , Al Shami S. , and Howell S. E. I. , Implementation of Teledentistry for Orthodontic Practices, Journal of the World Federation of Orthodontists. (2021) 10, no. 1, 9–13, 10.1016/j.ejwf.2021.01.002. 33642260

[10] Kılıç B. , İbrahim A. H. , Aksoy S. , Sakman M. C. , Demircan G. S. , and Önal-Süzek T. , A Family-Centered Orthodontic Screening Approach Using a Machine Learning-Based Mobile Application, Journal of Dental Sciences. (2024) 19, no. 1, 186–195, 10.1016/j.jds.2023.05.001. 38303845 PMC10829551

[11] Almishkha S. I. , Sulaimani O. M. , and Almutairi M. R. , et al.Precision Orthodontics in the Digital Era: AI, Teledentistry, and 3D Innovations for Individualized Treatment, Integrative Biomedical Research. (2023) 7, no. 1, 1–8.

[12] Lognay G. and Noortgate W. V. , Artificial Intelligence Innovations in Orthodontics: Enhancing Precision and Patient Outcomes, Annals of Orthodontics and Periodontics Specialty. (2025) 5, no. 1, 21–38, 10.51847/ppB9ggiYjW.

[13] Liedekerke L. V. , Lognay G. , Noortgate W. V.den , and Schaufeli W. D. , AI-Enabled Innovations in Orthodontics: Improving Treatment Precision and Clinical Results, Asian Journal of Periodontics and Orthodontics. (2025) 5, no. 1, 1–17, 10.51847/M4KUIR3e8o.

[14] Moeini A. and Torabi S. , The Role of Artificial Intelligence in Dental Diagnosis and Treatment Planning, Journal of Oral and Dental Health Nexus. (2025) 2, no. 1, 14–26, 10.61838/kman.jodhn.2.1.2.

[15] Bijoy L. , Gopal H. , Sriharini R. , and Kaushik K. , Artificial Intelligence in Orthodontics—A Narrative Review, International Journal of Environmental Sciences. (2025) 11, no. 12s, 172–182, 10.64252/0m2frt78.

[16] Albertina D. , Hasan A. , Gabriele T. , and Rasyid A. A. , Accuracy and Efficiency of Artificial Intelligence-Driven Treatment Planning in Clear Aligner Therapy: A Comparative Study With Conventional Methods in Bandung, Indonesia, Crown: Journal of Dentistry and Health Research. (2023) 1, no. 1, 38–51, 10.59345/crown.v1i1.55.

[17] Squires T. , Michelogiannakis D. , Rossouw P. E. , and Javed F. , An Evidence-Based Review of the Scope and Potential Ethical Concerns of Teleorthodontics, Journal of Dental Education. (2021) 85, no. 1, 92–100, 10.1002/jdd.12384. 32860244

[18] Chu S. B. and Ooi H. S. , The Digital Workflow in Dentistry: Adoption and Challenges, IIUM Journal of Orofacial and Health Sciences. (2025) 6, no. 2, 244–251, 10.31436/ijohs.v6i2.336.

[19] Gao S. , Wang X. , Xia Z. , Zhang H. , Yu J. , and Yang F. , Artificial Intelligence in Dentistry: A Narrative Review of Diagnostic and Therapeutic Applications, Medical Science Monitor. (2025) 31, e946676. 40195079 10.12659/MSM.946676 PMC11992950

[20] Kaushik R. and Rapaka R. , A Patient-Centered Perspectives and Future Directions in AI-Powered Teledentistry, Discoveries. (2024) 12, no. 4, 10.15190/d.2024.18, e199. 40109877 PMC11919542

[21] Kavousinejad S. , Ameli-Mazandarani Z. , Behnaz M. , and Ebadifar A. , A Deep Learning Framework for Automated Classification and Archiving of Orthodontic Diagnostic Documents, Cureus. (2024) 16, no. 12, 10.7759/cureus.76530, e76530. 39877794 PMC11774544

[22] Polizzi A. , Serra S. , Leonardi R. , and Isola G. , Clinical Applications of Artificial Intelligence in Teleorthodontics: A Scoping Review, Medicina. (2025) 61, no. 7, 10.3390/medicina61071141, 1141. 40731772 PMC12298516

[23] Surdu A. , Foia C. I. , and Luchian I. , et al.Telemedicine and Digital Tools in Dentistry: Enhancing Diagnosis and Remote Patient Care, Medicina. (2025) 61, no. 5, 10.3390/medicina61050826, 826. 40428784 PMC12113309

[24] Arksey H. and O’Malley L. , Scoping Studies: Towards a Methodological Framework, International Journal of Social Research Methodology. (2005) 8, no. 1, 19–32, 10.1080/1364557032000119616.

[25] Tricco A. C. , Lillie E. , and Zarin W. , et al.PRISMA Extension for Scoping Reviews (PRISMA-ScR): Checklist and Explanation, Annals of Internal Medicine. (2018) 169, no. 7, 467–473, 10.7326/M18-0850. 30178033

[26] Peters M. D. J. , Godfrey C. , McInerney P. , Munn Z. , Tricco A. C. , and Khalil H. , Aromataris E. , Lockwood C. , Porritt K. , Pilla B. , and Jordan Z. , JBI Manual for Evidence Synthesis, Scoping Reviews, 2020, JBI, https://synthesismanual.jbi.global. 10.11124/JBIES-20-00167 33038124

[27] Crowe M. , Sheppard L. , and Campbell A. , Reliability Analysis for a Proposed Critical Appraisal Tool Demonstrated Value for Diverse Research Designs, Journal of Clinical Epidemiology. (2012) 65, no. 4, 375–383, 10.1016/j.jclinepi.2011.08.006. 22078576

[28] World Health Organization , Global Oral Health Status Report: Towards Universal Health Coverage for Oral Health by 2030. Regional Summary of the Region of the Americas, 2023, World Health Organization.

