# Supplementary material for: Artificial Intelligence–Enabled Orthodontic Care for Remote and Underserved Populations: A Scoping Review of Access, Technology, and Public Health Integration
Source: Int J Dent. 2026 Sep 12;2026:3557207. doi: 10.1155/ijod/3557207 (PMC13570599; doi:10.1155/ijod/3557207)
Supplement: Supplementary file 1 [file IJOD-2026-3557207-s001.docx]

| Bibliographic Information | Study Design and Methodology | Type of AI Technology and Orthodontic Application | Population and Setting Characteristics | Key Findings | Public Health or Access-Related Outcomes |
| --- | --- | --- | --- | --- | --- |
| Sharma A et al. (2025), *Journal of Orthodontic science* | In vivo prospective clinical research. 40 patients divided into Control (Group A) and Test (Group B/AI monitoring). Evaluated Oral Plaque Index (OPI), Marginal Gingival Index (MGI), and White Spot Lesions (WSL) at baseline and intervals up to 3 months (P4). | AI-based remote monitoring system (Dental Monitoring® (DM)). Technology uses a smartphone app, scan box, and movement tracking algorithm. Application: Assessing and improving oral hygiene during fixed orthodontic treatment. | 40 patients scheduled for fixed orthodontic treatment. Divided into Group A (n=20) and Group B (n=20). Exclusion criteria: patients with physical limitations or those without dependable smartphone/internet connectivity. Setting: Department of Orthodontics, India. | Test group showed a substantial decrease in plaque score, gingival inflammation, and WSL compared to the control group. Integrating a remote monitoring system (DM) improves plaque, gingival inflammation, and WSL. | Dental Monitoring helped patients in retaining optimal oral health. DM can be very beneficial in isolated places with limited access to orthodontic care. Patients can reduce the time and expense of visiting the clinic in person. |
| Kılıç B et al. (2024), *Journal of Dental Sciences*. | Retrospective study. Evaluated the accuracy of a machine learning model on 524 pre-pubertal children and then on an independent validation dataset of 145 patients diagnosed by 5 clinicians. | Machine Learning (ML)-based mobile application. Application: Preliminary diagnosis of skeletal malocclusion (Class III vs not Class III; Class II vs Class I) using just one profile photograph. | 524 pre-pubertal children (aged 5–12 years, mean age 8.3). Included Class I, Class II Div 1/Div 2, and Class III malocclusions. | The final accuracy of the Class III vs not Class III model deployed to the mobile application was above 81%. The accuracy of the Class II vs Class I model was 69% (or 79.4% on independent validation). This study predicted skeletal class using profile pictures without medical apparatus. | The approach has the potential to improve access to orthodontic care, especially in underserved communities. It provides parents with important information about the orthodontic problem and the ideal age of treatment. Addresses the impracticality of screening all children worldwide over age 7. |
| Almishkha S. I. et al. (2023), *Journal of Angiotherapy*. | Systematic Review. Synthesized existing literature (peer-reviewed, industry reports) from between 2020 and 2022. | AI algorithms (Machine Learning, Deep Learning), Teledentistry, 3D printing. Application: Enhanced diagnostic accuracy, individualized treatment optimization, remote monitoring. | Review synthesizing data across multiple studies. Focus on individualized patient profiles. | AI improved diagnostic accuracy, individualized treatment planning, patient compliance, and reduced in-person appointments. | Teledentistry is particularly beneficial for underserved populations, facilitating equal access to orthodontic care. Digital workflows save 15–20% of costs compared to traditional methods. |
| Lognay G et al. (2025), *Ann Orthod Periodontics Spec*. | Narrative Review. Focused on current uses, challenges, and future prospects of AI in orthodontics. | AI (ML, neural networks, computer vision). Application: Predicting tooth displacement, individualized aligner design, continuous remote monitoring, automated cephalometric assessments (CNNs). | Narrative review covering different patient categories (children, adults, complex cases). | AI enhances diagnostic accuracy and efficiency. AI predictive systems achieved around 73% accuracy in estimating treatment outcomes (limitations remain for complex cases). AI simulates detailed tooth trajectories over time. AI aids early management in children (predicts malocclusion risks, identifies jaw anomalies). | Remote monitoring minimizes the frequency of clinic visits and expands access to orthodontic care. Tele-orthodontics benefits populations with limited access due to geography or cost. |
| Liedekerke LV et al. (2025), *Asian J Periodont Orthodont*. | Review. Summarized current AI applications, emphasizing precision and customized treatment. | AI (ML, computer vision, deep neural networks). Application: Personalized treatment plans, prediction of tooth movement, customized appliance design, remote monitoring. Uses CNNs for landmark identification and RNNs for movement forecasting. | Review covering diverse patient subgroups (young children, teenagers, older adults). | AI reduces overall treatment time. AI enables highly individualized treatment plans, optimizing appliance designs and force application based on unique anatomy. CNNs speed up cephalometric tracing and improve measurement consistency. Predictive orthodontics for early intervention is a future prospect. | AI makes orthodontic care more patient-centered, cost-effective, and widely available. Remote monitoring and instant feedback reduce unnecessary travel and enhance compliance. |
| Moeini A & Torabi S (2025), *Journal of Oral and Dental Health Nexus*. | Narrative Review using descriptive analysis. Focused on literature published between 2022 and 2025. | AI, ML, Deep Learning. Application: Automated cephalometric tracing, virtual treatment simulations, teledentistry/remote diagnosis, personalized treatment strategies. | Review synthesizing recent advancements in dental diagnosis and treatment planning (2022–2025). | AI automates cephalometric landmark detection with high accuracy, improving diagnostic efficiency and standardization. AI systems generate virtual treatment simulations and predict tooth movement patterns, facilitating precise planning. AI improves efficiency and reduces human error. | Teledentistry and remote diagnosis have expanded access to care. AI enhances diagnostic consistency and reduces treatment variability. |
| Lakshmi Bijoy et al. (2025), *International Journal of Environmental Sciences*. | Narrative Review. Explored historical evolution, core applications, advantages, and limitations of AI in orthodontics. | AI, ML, DL, CNNs. Specific tools: Dental Monitoring (DM), WebCeph, CephX, smart force/smart track. Application: Automated cephalometric analysis, malocclusion classification, outcome prediction, remote monitoring. | Narrative review summarizing AI applications in orthodontics. | AI can achieve more than 90% accuracy in landmark identification and reduce tracing time by over 75%. CNN model achieved >98% accuracy in image categorization (Ryu et al. cited). AI model for extraction decision reached 93.9% accuracy. Remote monitoring (DM) reduced in-office visits by 30–40%. | AI allows large-scale orthodontic screenings in epidemiological studies. Virtual consultations/apps offer tools valuable for initial screenings and triage. |
| Albertina D, (2023), *CROWN: Journal of Dentistry and Health Research*. | Retrospective study. Compared AI-driven planning vs. Conventional Methods (CM) in Clear Aligner Therapy (CAT) regarding accuracy (LII, PAR scores) and efficiency (planning time, number of refinements). | AI-driven software, Machine Learning. Application: Clear Aligner Therapy (CAT) treatment planning. | 100 patients treated with CAT in Bandung, Indonesia. CM Group (n=50), AI-Driven Group (n=50). Population: Age 18 years, mild to moderate malocclusion (Angle Class I or II). | AI-driven planning leads to significant improvements in efficiency. AI automates labor-intensive tasks (data processing, prediction). Enhanced accuracy translates to more predictable outcomes. AI optimizes treatment approach to minimize relapse risk and reduce treatment time. | AI enhances accessibility to care for a wider population. Remote monitoring extends the reach of orthodontic care to underserved populations, bridging geographical/socioeconomic barriers. |
| Squires T et al. (2021), *J Dent Educ*. | Evidence-based Review. Summarized the scope and ethical concerns of teleorthodontics based on four included clinical studies (up to Oct 2020). | Teleorthodontics, with reference to AI monitoring via Dental Monitoring. Application: Remote treatment planning and monitoring. | Review synthesizing 4 clinical studies. | Teleorthodontics facilitates treatment planning/monitoring by sharing records. DM allows monitoring of tooth movement and treatment progression using at-home digital photographic technology. Reduces the need for in-office patient visits. | Teleorthodontics facilitates access to care for patients in remote/rural geographic locations. May be cost-effective by reducing expenditures such as transportation and direct consultation. |
| Olawade D. B. et al. (2025), *Dent. J.*. | Narrative Review. Focused on current applications, future prospects, and challenges of AI in orthodontics. | AI, ML, Neural Networks, Computer Vision. Uses CNNs (landmark detection) and RNNs (movement prediction). Application: Personalized treatment planning, prediction, custom aligner fabrication, real-time monitoring. | Review summarizing benefits across diverse patient categories (children, adults, severe malocclusion). | AI-enhanced diagnostic tools showed higher accuracy (p < 0.05). AI-assisted planning reduced overall treatment time by an average of 4.3 months. AI analysis time reduced by as much as 80-fold compared to manual methods. Meta-analysis cited reported overall accuracy of AI treatment planning as 95.47%. | AI-powered remote monitoring decreases the need for in-person visits, making orthodontic care more accessible. Tele-orthodontics could expand into a fully remote experience, beneficial in underserved areas. |
| Chu S. B. & Ooi H. S. (2025), *IIUM Journal of Orofacial and Health Sciences*. | Opinion Article/Review. Focuses on digital workflow adoption, advantages, and challenges. | Digital technologies (IOS, CBCT, CAD/CAM, 3D printing). AI integration discussed as a potential future direction. | General dental practices. | Digital technologies result in greater diagnostic accuracy, enhanced patient outcomes, and streamlined operations. Integration of IOS, CBCT, and facial scans enhances case assessment and visualization for reliable treatment planning. | Digital technologies improve patient communication. Challenges include elevated expenses and steep learning curves for practitioners. Need to ensure privacy and security of patient data. |
| Gao et al. (2025), *Med Sci Monit*. | Narrative Review. Examined AI progress across dentistry domains, focusing on enhancing diagnosis and optimizing treatment. | AI, ML, ANN, Deep Learning. Application: Automated landmark detection/cephalometric analysis, treatment planning (extraction decision, orthognathic surgery planning). | Review spanning general dentistry applications. | Neural network model for extraction vs non-extraction achieved 93% accuracy. Deep learning achieves accuracy comparable to experienced orthodontists in cephalometric analysis, significantly reducing workload and human error. AI provides guidance for treatment planning, especially for less experienced orthodontists. | AI aids in prevention, early screening, and follow-up monitoring. AI integration into teledentistry improves access to cancer diagnostics in underserved populations (general finding). |
| Kaushik R et al. (2024), *Discoveries (Craiova).* | Scoping Review following Arksey and O’Malley framework. Focused on patient-centered perspectives and implementation challenges. | AI, ML, DL, CNNs. Application: AI-powered teledentistry, Diagnostics, Personalized treatment planning, Remote monitoring. | Review of studies on AI in teledentistry, focusing on patient outcomes and underserved populations. | AI improves diagnostic accuracy and treatment planning. AI algorithms excel in analyzing dental images and identifying issues. Predictive analytics helps plan preventive follow-up procedures. AI enhances communication and patient satisfaction. | AI increases the availability of dental care to underserved populations. Remote monitoring allows timely interventions without frequent in-person visits, benefiting rural/limited mobility patients. Reduces healthcare disparities. |
| Kavousinejad S et al. (2024), *Cureus*. | Study involving the development and validation of a deep learning framework. Methodology: Sequential classification approach, internal and external validation (13,729 new samples). | AI-driven deep learning framework based on Convolutional Neural Networks (CNNs). Application: Automated classification and archiving of orthodontic diagnostic images (13 distinct categories, including radiographs). | Dataset of 61,842 images collected from three dental clinics in Tehran, Iran. Patients aged 7 to 40 years. | The model achieved a mean accuracy of 99.23% during external validation. The AI framework enhances workflow efficiency and reduces human errors in classification and archiving. Establishes a foundation for future automated diagnosis and treatment planning. | Automating image classification streamlines processes, saving time for both dentists and patients. Reduces the workload of healthcare professionals. |
| Polizzi A et al. (2025), *Medicina*. | Scoping Review. Search up to April 2025. Based on nine included studies. Focus on real-world clinical applications. | AI, Deep Learning, CNNs (primarily Dental Monitoring™ (DM)). Also mentions StrojCHECK™ (decision tree algorithm). Application: Teleorthodontic monitoring (aligner fit, tooth movement, hygiene, GO/NO-GO progression). | Orthodontic patients of any age undergoing remote monitoring. Review synthesizes 9 studies. | Reported benefits included reduced in-office visits (up to 33%). DM system allows for successful monitoring of aligner fit and complex movements. AI-based active reminders improved oral hygiene (statistically significant reduction in indices). DM AI detection of oral hygiene issues had low sensitivity but high specificity (tendency to underreport problems). | AI-powered systems enhance treatment efficiency and patient engagement. AI triage systems are a promising future avenue to improve access by enabling asynchronous consultation and early case sorting. |
| Surdu A et al. (2025), *Medicina*. | Comprehensive Review of existing literature and case studies. Explored applications, benefits, and challenges of teledentistry. | AI-assisted diagnostic systems. Also addresses general Teledentistry tools (digital imaging, EHRs, consultation platforms). Application: Remote consultation, telediagnosis, AI-assisted diagnosis. | Review spanning multiple dental disciplines. | Teledentistry facilitates early disease detection, reduces wait times, and minimizes unnecessary visits. AI systems aid in assessment of treatment course and retention. AI-driven remote monitoring improves oral hygiene. | Teledentistry enhances access to oral healthcare by enabling remote consultation, diagnosis, and patient management, especially for remote and underserved populations. |
| Elnagar M. H. et al. (2025), *Journal of the World Federation of Orthodontists*. | Study aimed to evaluate the validity and usability of DM. Used a study flow diagram (N=104 subjects enrolled). | Artificial Intelligence Driven Remote Monitoring (AIDRM) system, DentalMonitoring (DM). AI driven by CNN-based architectures. Application: Providing standardized, high-quality photo sets for visual assessment of clinical parameters (appliance integrity, hygiene indicators). | N=104 subjects enrolled. Setting: Multi-center private orthodontic practices and academic setting in the USA. | DM AI demonstrated high agreement (98.1% to 99.5%) with clinical assessment for detecting appliance issues (e.g., attachment, bracket, self-ligating clip). DM is consistently capable of generating high-quality image sets without requiring patient assistance or prior experience. | Opens the door to a new paradigm in orthodontic patient follow-up and new dental prevention and treatment follow-up strategies. |
| Snider V et al. (2024), *Am J Orthod Dentofacial Orthop*. | In-vivo prospective clinical study. Compared DM AI detection to clinical examination (Gold Standard). | Artificial Intelligence Driven Remote Monitoring (AIDRM), Dental Monitoring (DM). AI image analysis and oral hygiene notification algorithm. Application: Evaluating DM AI accuracy in identifying oral hygiene (plaque/calculus, gingivitis, recession). | 24 patients monitored undergoing fixed orthodontic treatment. Followed for 10-13 months (10 adjustment visits/time points). Setting: University of Illinois Chicago’s orthodontic clinic. | DM AI detection showed low sensitivity (0.22 to 0.53) and high specificity (0.94 to 0.99) across plaque/calculus, gingivitis, and recession detection. This indicates that the DM AI algorithm has a tendency to underreport the presence of oral hygiene conditions. | Telemonitoring applications (such as DM) are becoming increasingly used in our digital world for remote treatment monitoring. |
| Snider V et al. (2023), *Orthod Craniofac Res*. | In vivo prospective clinical study. Compared DM Group (n=24) monitored by weekly scans/notifications vs. Control Group (n=25). Measured Plaque Index (OPI) and Modified Gingival Index (MGI). | Dental Monitoring™ (DM™) Artificial Intelligence Driven Remote Monitoring Technology (AIDRM). Uses AI-based personalized active notifications. Application: Improving oral hygiene during fixed appliance treatment. | DM Group (n=24) followed for 13 months, Control Group (n=25) followed for 5 months. Fixed orthodontic brackets. Setting: University of Illinois Chicago's orthodontic clinic. | The DM group had statistically significantly lower OPI and MGI values (OPI=1.96, MGI=1.56) than the control group (OPI=2.41, MGI=2.17) after 5 months. AIDRM via weekly DM scans and personalized active notifications may improve oral hygiene over time in orthodontic patients. | AIDRM helps individuals take more agency over their oral health and improves communication between the patient and their orthodontist. |
| Homsi K et al. (2023), *Am J Orthod Dentofacial Orthop*. | In-vivo prospective clinical study. Tracked 24 patients across 13.4 months. Compared DM remotely generated 3D models to iTero Element 5D IOS models. | Dental Monitoring (DM) Artificial Intelligence Driven Remote Monitoring (AIDRM) technology. Uses AI algorithms, machine vision. Application: Tracking tooth movement and reconstruction of 3D digital models during fixed appliance treatment. | 24 patients (aged 14-55 years). Fixed orthodontic treatment with labially affixed brackets/bands. Average study period 13.4 months. Setting: The University of Illinois Chicago, Department of Orthodontics’ clinic. | DM AI tracking algorithm can track tooth movement and reconstruct 3D digital models to a clinically acceptable degree for orthodontic application. No clinically significant difference found in global deviation between DM reconstructed models and iTero IOS models. | Teleorthodontic applications eliminate unnecessary appointments, improve outcomes, and provide potential for cost savings and closer monitoring. |
| Surovková J et al. (2023), *Appl. Sci.*. | Report of an assessment over a period of three years, describing the concept of a novel AI-powered orthodontic workflow. | AI-powered telehealth solution (Dental Monitoring® (DM)). Uses AI and knowledge-based algorithms. Also discusses AI-language models (ChatGPT 4). Application: Automated assessments of treatment progress, quality assessment, and individualized appliances design. | Orthodontic practice assessment over a three-year period; focused on clear aligner treatment. | AI improves dental practice through more precise and personalized treatment. Providers are alerted immediately when patient progress deviates. DM tracks clear aligner fit/retention and is suitable for all clear orthodontic modalities. | AI implementation creates new roles and responsibilities for trained dental assistants and nurses. DM increases treatment effectiveness through increased permanent control. Raises new ethical and legal issues. |
| Park J. H. et al. (2021), *J Clin Pediatr Dent*. | Review Article. Provided information on HIPAA-compliant teledentistry systems for orthodontists considering remote practice. | Artificial Intelligence (AI) assisted treatment monitoring (Dental Monitoring). DM uses AI to track tooth movement and detect complications remotely. | Orthodontic patients/practices. | DM verifies and tracks the movement of each tooth for fixed appliances and clear aligners. DM can alert providers of common conditions (broken brackets, unseated aligners). Dental Monitoring is the only platform reviewed that has the capability of full AI-assisted monitoring. | Teledentistry platforms enable patients to request, receive, and transmit information irrespective of time and place. Helps minimize in-person interactions, crucial during pandemics. |
| Park J. H. et al. (2021), *Journal of the World Federation of Orthodontists*. | Featured Review Article. Discussed types of teledentistry, implementation guidelines, and regulatory considerations. | Artificial intelligence (AI)-assisted treatment monitoring (Dental Monitoring). Application: Tracking orthodontic movements and detecting orthodontic treatment complications remotely. | Orthodontic practices. | AI is used to help track orthodontic movements and detect orthodontic treatment complications remotely using patient-taken intraoral photos. Telecommunication is useful for consultations, reviewing forms, monitoring appliances (expanders, aligners), and guiding minor emergencies. | Online checkups are more time-saving and economically appealing to both clinicians and patients. Teledentistry expedited adoption due to patient demand and the need for social distancing (COVID-19). |

Supplementary Table 1: Data Extraction and Charting: AI in Orthodontics
